# Nutritional and end‐use perspectives of sprouted grains: A comprehensive review

**DOI:** 10.1002/fsn3.2408

**Published:** 2021-06-23

**Authors:** Ali Ikram, Farhan Saeed, Muhammad Afzaal, Ali Imran, Bushra Niaz, Tabussam Tufail, Muzzamal Hussain, Faqir Muhammad Anjum

**Affiliations:** ^1^ Department of Food Science Government College University Faisalabad Faisalabad Pakistan; ^2^ University Institute of Diet & Nutritional Sciences The University of Lahore Lahore Pakistan; ^3^ The University of Gambia Serrekunda Gambia

**Keywords:** factors affecting, germination, nutritional changes, products, sprouting

## Abstract

Scientific literature is evident that the germinated seeds possess a promising potential for essential nutrients, flavors, and textural attributes over nongerminated grain. In recent decades, sprouting has also been investigated as a potential green food engineering technique to boost the nutritive profile of grains. Sprouting grains have multifold applications in different fields such as baking, pharmaceutical, and cosmetic industries. During sprouting, shifting of molecular structures to macroscopic takes place. Sprouting reactivates the grain metabolism which leads to the catabolism and degradation of antinutrient and macronutrient compounds. These modifications have an effect on human health and on the nutritional content of the foodstuffs. Sprouting grains have high bioactivity against diabetes and cancer. Germination is also an outstanding green food development technique to increase the seed nutritive profile in terms of quality. The present review focuses on the sprouting of grains, changes in nutritional profile, and the technological exploration of sprouted grains.

## Graphical abstract

Sprouting grains has multifold applications including nutraceutical, functional foods, pharmaceutical, and cosmetic industries. During sprouting, a shift of molecular structures to macroscopic takes place. Sprouting reactivates the grain metabolism which leads to the catabolism and degradation of antinutrient and macronutrient compounds. These modifications have an effect on the health and on the nutritional content of the foodstuffs.

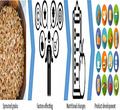

## 1 INTRODUCTION

Cereals are a part of the grass family named *Gramineae*. They grow dried, single‐seeded fruits termed grains, consisting of seed and a fruit coat (pericarp). The grain itself contains the endosperm, embryo (germ), nuclear epidermis, and also the coat of the seed. Chemical compositions of cereals are put into the cell walls or even other barriers in components isolated from each other (Delcour et al., 2010).

Pulses have long been considered to be an excellent source of proteins, fiber, and antioxidant compounds, such as phenolic acids, flavonoids, and polyphenols, for their nutritious and health‐promoting attributes and also have a lower glycemic index (Hall et al., 2017).

Therefore, in many food recipes formulated for the general public or in particular for special diets like vegetarian, and gluten‐free, the pulse may be taken as an entirety and being used as an element. Additionally, the pulses consumed by consumers are unexplored by the prevalence of antinutrients, including trypsin inhibitors, phytic acid, and some indigestible oligosaccharides, that cause digestive distress (Hall et al., 2017). Last but not least, the presence of nonflavors can discourage the usage of pulses when used in food processing (Roland et al., 2017).

In this regard, many processes have been implemented to pulses—such as soaking, extrusion, dehulling, and fermentation—in the light of scientific proof to suggest an increased intake of pulses to boost the health (Patterson et al., 2017). Sprouting is very common in the marketplace among such technological processes and constitutes a revival of the trend in healthy foods because of the positive effects on improved nutrient composition (Ghavidel & Prakash, 2007) and tastes (Roland et al., 2017).

For many years, sprinkled grains have generally been designated as a seed with a noticeable radical or as a foodstuff centered on the common conviction that they offer essential nutritive, flavors, and texture advantages over nonsprouted grain equivalents. In recent decades, sprouting has also been investigated as a potential green food engineering technique to boost the nutritive profile of grains and also to analyze secondary metabolites with diverse uses in the nutraceutical, functional foods, pharmaceutical, and cosmetic industries. During seed germination, there are various shifts, varying from molecular structures to macroscopic ones (Lemmens et al., 2021).

Sprouting reactivates grain metabolism which leads to the catabolism as well as degradation of antinutritient and macronutrient compounds and to secondary metabolites synthesis pathway that has potential benefits for health. These modifications have an effect on the health and on the nutritional content of the foodstuffs (Peñas & Martínez‐Villaluenga, 2020).

Sprouted beans serve as an ingredient for several years on account of the overall conviction that their unsprouted or sound grain equivalents have major nutritional, tasteful, and textured advantages. In the assessment of the advantages of sprouting seeds, two factors should be considered and how the advantages differ among research and records, such as (a) how production methods make contributions to the variations and (b) how much more the genetic structure of the precursor material is variable. This is the clear factor about the dietary improvements in the Omary et al. (2012) research.

Recent research shows that the nutritive assessment of grains can be increased by increasing the amount of free A.A, simple sugars, bioactive products, and organic acids (Wang et al., 2005). They can also decrease inhibitory factors for antinutrition and digestion, for example, lectin, and protease inhibitors (Aguilera et al., 2014). Sprouting grains also have revelation biological functions, for example, antidiabetics antioxidants and anticancer. Germination is also an outstanding green food development technique to increase the seed nutritive profile. Increases protein and starch digestibility in the seed and also the quality of nutrients including vitamins and amino acids. The germination, therefore, enhances the nutritional value and edible grain quality (Mikulinich & Guzikova, 2021). Various studies have shown that germinated grain intake may decrease.

## 2 METHODS

The literature search was carried out using scientific databases comprising Scopus, Science Direct, PubMed, Cochrane Library, Science Hub, and Google scholar, using the following subject headings sprouting and germinated cereal using keywords: "sprouting grains, germinated grains, nutritional changes, factors affecting the sprouting, and products prepared." The authors also obtained data from primary and secondary sources as well.

## 3 GERMINATION/SPROUTING PROCESS

Sprouted seeds are developed by a managed germination process supervised by the producer to deliver the appropriate product, batch after batch. As sprouting is a complicated process, it is crucial to understand the occurrences of the grain and how the method is changed based on the seed and cultivation condition types to produce a higher quality product.

The kernels must meet the minimum moisture of 35%–45% and a lower temperature of 4℃ to start the sprouting of wheat (Gooding, 2009). Water moves through the micropyle into the wheat kernel, where it reaches the scutellum and germ to start sprouting (Rathjen et al., 2009) and proceeds to travel across the kernel, accruing among the seed coat and pericarp (Rathjen et al., 2009). If the humidity reaches the least level, the seed starts to release or/and synthesize plant hormones including abscisic acid, ethylene, and gibberellic acid. The release of these hormones in the grain stimulates the production of amylase, humiliating enzymes, lipase, and proteases.

The breakdown of protein, starch, and lipids by such enzymes supplies the growing embryo with an energy supply, although this breakdown can have a substantial effect on the efficiency and consistency of the ingredient. The rise in amylase would result in a decline in the peak viscosity of the starch pasting, which affects the ingredient's starch activities. The raise in proteases can contribute to the possible breakdown of proteins that form gluten, thus decreasing the overall consistency of the dough. The rise in lipase enzymes will contribute to lipid breakdown as well as the high potential for auto‐oxidation, resulting in the end product developing off‐flavors (Marti et al., 2020).

Two different types can be put on the industry for sprouted foods: in either wet‐mash type, wherever the substance is processed frozen, or as a dry product, delivered either as flour or dry sprouted seed. The first two stages are the same irrespective of whether the substance is a moist mash or a dry one: steeping and germination. The seeds are immersed in liquid during steeping, till they meet the optimal moistness content. All additional water is discharged and the seed is allowed to sprout until the correct moisture is obtained. In a variety of different kinds of vessels, germination may occur. A sprouting bed, comparable to those used in the malting sector, is a traditional vessel. Despite the germination vessel, to maintain a uniform germination percentage across the content, the germinated seeds need to be maintained in an aerobic atmosphere with steady in addition to continuous airflow and high temperature. The wet‐mash content is automatically refined into the final end‐ingredient until germination is finished, or it is preserved for use at a later stage. At varying temperatures and times that are specific to each grain form and producer, drying or kilning will continue. These set points are optimized, product separation patented, and quality continuity reached (Finnie et al., 2019).

Much attention has been paid to Sprouted Grain. For sprouting, almost any cereal and leguminous seed are eaten by individuals as a food source (wheat, rice, maize, sorghum, barley, millets, rye, oats, mug beans, soybeans, and black beans), has been included. Among them, germinated barley, wheat, sorghum, oat, and beans are the most commonly used sprouted grains. Several authors have reported on these sprouted grains' nutritional properties, processing applications, and physiological functions.

## 4 FACTORS AFFECTING THE SPROUTING CONDITION

### 4.1 Germination environmental conditions

For productive seeds to germinate and produce sprouts, these factors have to be desirable. These requirements involve:

1. A sufficient water supply.
2. A suitable temperature.
3. A particular mixture of atmospheric gases.
4. Light (specific seeds).
5. Exclusion of germination inhibitors.

Basic criteria for these factors differ with varieties and species.

#### 4.1.1 Water

During germination, the first process is the water absorption by the seed, which is mainly affected by the seed's permeability. However, both the seed composition and the accessibility of water in the environment and the solution solute concentration will also affect the physical process of this imbibition. The viability of the seeds of either dead or alive seeds is unrelated to Imbibition results in a major pressure growth, which is of interest during the development of germination and sprout as it can split the seed coat. It is also followed by heat released when the water is first absorbed. The extent of this imbibition pressure reflects the water retention capacity and the quantity of water available for seed hydration during sprouting and subsequent germination. Imbibition lessens due to osmotic effects with increased water solute concentration. It is primarily a protein that absorbs the germination water. Cellulose also will contribute to the inflammation, but the starch will not swell significantly in the seeds, even if the amounts of cereal grain are rather high. Only a change of heat, acid, or enzymes to the starch will result in considerable swelling, but conditions do not occur during germination in grain kernels.

In general, protein is higher in wheat than maize, and therefore, more water is expected to absorb in germination. In the sprouting of cereal grains, controlling the water supply is extremely important. The most important factors in producing sprouts are appropriate moisture and temperature conditions.

#### 4.1.2 Temperature

Average temperatures of germination vary with various grains of cereal. The temperature and the variety of seeds at which it germinates are dependent on the species, genetic variations, the varieties, the source of the seed, and its age. Typically, the optimum temperature is below and above, which slows but does not preclude germination. The optimum temperature is the one that achieves the maximum germination rate in the shortest time possible. Germination of all seeds is resisted at extremely high and very low temperatures. Optimum germination temperatures typically range from 20 to 30℃. There have been just a few optimum temperatures above 30℃. In the optimum definition, the maize and rice level is about 35℃ taking into account both the high final germination percentage and germination pace. Minimal seed germination temperatures are difficult to estimate since long incubation times can be required at the coldest temperature where the germination may happen. If germination does not occur within a certain time during incubation at low temperatures, so prolonged incubation at that low temperature does not lead to germination. However, not all germination failures should be assigned to an incubation that is too short." Most seeds need to be subjected to certain temperatures before being placed at a temperature that is favorable for germination or sprout. Seeds are then handled at high or low temperatures that do not encourage sprouting.

#### 4.1.3 Atmospheric condition

The quality of the atmospheric air influences seeds germination. In an atmosphere of 20% oxygen and 0.03% carbon dioxide, most seeds will germinate the natural amounts of these gases in the air. However, certain cereal grains exhibit increased germination when the oxygen stress is greater than the usual 20%. As germination requires energy expenditure, this is predicted and energy‐requiring processes are typically oxidation processes.

The effects of carbon dioxide are the inverse of the germination of oxygen. Many seeds fail to germinate if the stress of carbon dioxide increases considerably. However, a very high concentration of carbon dioxide, which inhibits germination, has a beneficial impact on grain storage. Rice appears to have an adaptation of germination under anaerobic conditions, but this is not a sufficient condition for the germination of rice. During sprouting in a container, the composition of the atmosphere around the seeds will change, leading to a decrease in oxygen content and elevated carbon dioxide levels. Germination and sprout growth are normally improved by enhanced and regular ventilation to ensure optimum atmospheric conditions in sprout output.

#### 4.1.4 Dark light

Several plants display variability in behavior toward the light: roses, some vegetables. Although some of these plants only germinate in the dark, others need constant light exposure. But the seeds of crops, like the cereal grains, also germinate in the dark and the sun. There is no evidence that light affects germination and cereal blossoming. Cereals have successfully blossomed in both the presence and absence of light.

#### 4.1.5 Inhibitors of germination

At toxic concentrations, certain chemical compounds that are toxic to living organisms will also inhibit germination. Germination inhibition may also be attributed to compounds that are believed to interact with certain active metabolic processes throughout germination. Herbicides, as well as insecticides, can have certain consequences and chemical agents used as seeds for the next crop year to protect kernels of cereal grains. For these purposes, only untreated grains can be used for germination.

## 5 NUTRITIONAL MODIFICATIONS AFTER SPROUTING

During germination or malting, many biochemical modifications occur, impacting product possessions such as shape, bioactivity, consistency, flavor, and digestibility (Katina et al., 2007; Salmenkallio‐ Marttila et al., 2001). Germination induces enzymes to split up carbohydrates, lipids, and proteins into basic methods and stimulates proteases involved in destroying proteins, thus enhancing the bioavailability of nutrients. Starch and proteins are activated and decomposed by hydrolytic enzymes, resulting in a rise in oligosaccharides as well as amino acids in the oat (Mikola et al., 2001), wheat (Yang et al., 2001), barley (Rimsten et al., 2002), and rice (Manna et al., 1995).

### 5.1 Carbohydrates

Since the mobilization of complex polymers, such as starch, is one of the most studied processes for seedling growth, significant changes in seed carbohydrates have been thoroughly studied in many of these sprouted grains (Aoki et al., 2006; Gujjaiah & Kumari, 2013). Amylases catalyze the starch hydrolysis, preserved as amylopectin and amylose, into simple sugars in germinating grains, that is, the reduction of glucose and maltose sugars and, to a lesser degree, nonreducing sucrose sugar (Aoki et al., 2006), resulting in increased digestibility (Chung, Cho, & Lim, et al., 2012; Chung, Cho, Park et al., 2012; You et al., 2016). (Agu et al., 2012). At the germination temperature of 20°C (Chiba et al., 2012), 5‐day old buckwheat shoots exhibited glucose to maltose proportion of 3.5:1; this ratio appeared to be related to the concentrations of α‐amylase and β‐amylase produced in cereals throughout malting (Scofield et al., 2007). In response to germination time, differential effects were also identified, with sucrose to be governing carbohydrate source throughout the initial germination process of wheat and glucose as well as maltose even during later phases (3‐day postimbibition) (Noda et al., 2004); sucrose also predominated shortly after imbibition in rice. However, for a long postimbibition period, germinated rice seedlings showed elevated sucrose levels, glucose content raised faster, and maltose did not occur at a substantial level till much far ahead (7‐day postimbibition). Sprouted seed tissues also play a key role: Higher levels of glucose were found in wheat and rice endosperm compared to sucrose in scutellum (Gujjaiah & Kumari, 2013). However, the dynamics of the transport system for the absorption of remobilized carbohydrate reserves through the endosperm scutellum differ slightly between the different plants. The mechanisms of starch hydrolysis are relevant to the form and position of starch granules and the components of starch. In general, starch hydrolysis results in minor changes in the amylose material, as has been seen in many plants, including wheat (Noda et al., 2004).

### 5.2 Protein

Usually, the crude protein of various cereals varies from 8% to 16% of dry mass (dm) (Donkor et al., 2012). Sound seed crops have low concentrations of intrinsic peptidase behavior, significantly increasing during one day of growing (Abd Elmoneim & Bernhardt, 2010; Faltermaier et al., 2015). barley, In sorghum, oats (Abd Elmoneim & Bernhardt, 2010), wheat (Faltermaier et al., 2015; Seguchi et al., 2010), and rye (Mäkinen & Arendt, 2012), a two‐ to fivefold rise in peptidase activity was detected while sprouting at 15–27℃ for 3–7 days. During sprouting, endopeptidases are formed and concealed from the layer of aleurone and scutellum. They are important for the growth of seedlings as they break down the stored proteins that enable functional proteins (e.g., β‐amylase) (Faltermaier et al., 2015). At 40–50℃ and pH 3.5–6.5, endopeptidases have optimum activity (Schwalb et al., 2012). Proteolysis is extra severe after prolonged germination periods (lowest possible 2 days) and at extreme temperatures (20–28℃) (Cáceres et al., 2014). In general, this does not create serious changes in total protein content when sprouting results in protein hydrolysis (Cáceres et al., 2014; Świeca & Dziki, 2015).

However, a substantial reduction of 3%–10% of the protein in germinated rice, wheat, and sorghum has been documented by some studies. In comparison, in sprouted barley, new researchers have recorded an upsurge in protein (5%–10%). The decline in protein was due to the leaching in the steep water of water‐soluble peptides (Afify et al., 2012), whereas the rise can be clarified by respiratory loss of carbohydrates. However, comparative variations in protein between sprouted or nonsprouted cereals were reported to be less than 10%, suggesting that sprouting typically does not have a major effect on the total protein content. Sprouting brown rice and wheat at 20–25℃ for 3–7 days does not substantially alter albumin and globulin levels (less than 35 kDa). Though, an improvement in albumins, high in amino acids, has also been reported once growing oats. It can be concluded that during sprouting, the oat preservation proteins are damaged and denatured. Besides, malting wheat has also been observed to induce a threefold change in protein levels that can be extracted from water (12–44 kDa). In particular, this increase appears mostly during 1 and 2nd sprouting days. It may also be influenced by prolamin, globulin, and glutenin partial degradation. In addition to peptides with varying MW, the storage proteins are degraded into FAA, the content of which up to a ratio of 5–10 when sprouting brown rice, wheat (Ohm et al., 2016), oats, and sorghum at 13–30℃ for 3–5 days. Phenylalanine particular, there was a high increased level of the basic amino acids lysine, leucine, isoleucine, threonine, and valine. As a result of sprouting, both the solubility and digestibility of proteins are greater. In this respect, when sprouted at 17–27℃ for 3–5 days, a 1.2‐ to 2‐fold rise in protein solubility has also been observed in barley as well as in sorghum (Afify et al., 2012). The digestibility of protein augmented from 34% to 55% in millet when sprouted at 30℃ for 4 days and from 50% to 65% to 65% to 80% in sorghum (Abd Elmoneim & Bernhardt, 2010; Afify et al., 2012) and barley when sprouted at 22–27℃ for 3–6 days. In comparison, no improvements in protein digestibility were observed (Świeca & Dziki, 2015) when sprouting wheat at 20–25℃ for 4 days. This was linked to a rise in the number of free phenolic during sprouting, which can shape and reduce the digestibility of insoluble protein complexes. Several phenolic components can also adversely inhibit the occurrence of the digestive tract enzymes and their substrates' affinity (Świeca & Dziki, 2015).

### 5.3 Lipids

Lipids abound in living muscles of whole grains, that is, embryo, sauce, and aleurone, as oil, even in cereals wherever starch is the primary carbon in the endosperm (triacylglycerols, TAG). Coordinated metabolic activity, which begins from germination, involves the mobilization of TAG by oil bodies, contributing to the total transformation of oil to sugar (Graham, 2008). First of all, the lipases release TAG's esterified fatty acids (FAs). Via the β‐oxidation and glyoxylate processes can be depleted and later converted into sugars. Additional data can be retrieved from the published literature on the paths complicated in transforming TAG into sugars in seed germination. In oats, which is unusual among cereals because of its great oil content relative to starch as well as protein concentration, the squalor of embryo oil reserves was observed earlier than that of scutellum oil reserves (Leonova et al., 2010); the mobilization of endosperm TAG reserves taking place later, 1–2 days after imbibition, as well as coincided with FFF accumulation. In waxy wheat, sprouting during 48 hr of germination did not considerably affect the FA confirmation of both free as well as bound lipids and the quality of essential FAs (linoleic as well as linolenic acids). As noted by (Ozturk et al., 2012), the linolenic acid (18:3 n3) concentration increases, whereas the number of cis‐18:1, as well as cis,cis‐18:2 FAs, reduced, Sprouting had a major impact on the FA formulation of 9‐day old wheat seedlings. Conversely, linoleic acid, palmitic acid, and oleic acid were the FAs most expressed in grain sprouts after 3 days of germination (Márton et al., 2010). During seedling growth, lipid catabolism supplies carbon and energy sources required for biochemical and physicochemical alteration. However, there are already high levels of lipase activity in normal mature oat grains that stay unaffected or even decrease during propagation, which is unusual surrounded by cereals (Mäkinen & Arendt, 2012). As a consequence of lipase activity, an 8%–15% reduction in lipid content exists in millet sprouted at RT for 3 days, in barley sprouted at 22℃ for 5 days, and in oat germinated at 16℃ for 6 days. The increase in sprouting conditions contributes to a greater breakdown of lipids. A decline in the total lipid of 18%–28% was reported in millet sprouted at 32℃ for 2 days, in wheat sprouted at 30℃ for 2 days, and in rice bloomed at 25–30℃ for 1–5 days. Destruction of triglyceride levels to monoglyceride and FAA by lipase catalyzes (Kubicka et al., 2000). Glycerol, as well as FAA, is primarily changed into sugars, which are being led to the rootlet and the shoot for use in the scutellum.

### 5.4 Phytate and minerals

Changes in minerals before and after sprouting are shown in Table 1. Phytate has a high concentration in various herbal food products and constitutes the key source of phosphorus storage in ripe grains and legumes. However, the poorly endogenous phytase in humans restricts phosphorous use (Ozturk et al., 2012), as it is characterized by high affinity to cations and thus deemed an antinutrient factor, has a negative effect on the bioactivity of mineral elements, including Ca^2+^, Fe^2+^, Zn^2+^, Mg^2+^, Cu^2+^, and Mn^2+^, as it is categorized by strong cation chelation. Phytases include the denaturation of phytate into Myo‐inositol as well as ortho‐phosphate and the inorganic phosphate of the phytases of high‐molecular‐weight phosphatase histidine acid. At the start of the sprouting process, phytase activity is extremely low and in the first few days, it increased to eightfold in barley (Boukid et al., 2017). However, the phytase concentration in whole grains varies significantly with the maximum grains and the lowest grain oats. As a result, throughout germination phytate content is reduced to another level. Sprouting of rice over 12–72 hr resulted in a 60% reduction in phytate content while degradation of phytate by up to 87% was observed in four‐day‐old sorghum seeds. Additional studies have been carried out on the degradation of phytate throughout plant growth of brown rice, barley, Perl millet, maize, sorghum, and wheat (Faltermaier et al., 2015; Seguchi et al., 2010). The bioaccumulation of phosphorus and minerals is growing as phytate content reduces. Germination has contributed to lower levels of Ca in barley and also in wheat and rising Mg in oats, barley, or wheat. In maize's, the content of the main macroelements (K, Ca, Na, Mg, and P) decreased following the two days of sprouting and increased until 6 days, minor minerals (Zn, Fe, Cu, Mn, and Co) (Chiba et al., 2012). Increased HCl extractability of the major and trace minerals was observed for these species during sprouting, although the cultivar was heavily influenced. The extractability of Ca, Fe, and Zn in whole grain rose to 90.2 by 76.9%, 18.1%, and 65.3%, respectively, 37.3% and 85.8%, following 96 hr of finger millet germination. In 2012, Zn, as well as Fe bio‐accessibility, increased from 15% and 14%, respectively, in sprouted wheat to 27% and 37% in the sprouted wheat processed by hydrothermal processes (Afify et al., 2012).

**TABLE 1:** Changes in mineral before and after sprouting

Cereals	Mineral	Content		References
	Mg/100 g	Before	After	
Amaranth	Ca	200	228	Gamel et al. (2006)
	Zn	3.9	4.8	
Buckwheat	Ca	56	79	Lee et al., (2004) and Omary et al. (2012)
	P	1,086	1,269	
	Mg	519	609	
	K	552	658	
	Na	1.2	3.2	
	Fe	4	4.6	
	Zn	6.1	7.6	
	Mn	7.2	8.4	
	Li	0.02	0.005	
	V	0.03	0.05	
	Cr	0.095	0.11	
	Cu	0.73	0.86	
Millet	Ca	36	65.7	Mbithi‐Mwikya et al. (2000) and Mamiro et al. (2001)
	Fe	19.4	34.9	
	Zn	42.2	28.8	
	Cu	35.2	56.5	
	Mn	52.8	59.9	
Quinoa	Fe	8.43	8.57	Omary et al. (2012)
	Mn	2	1.87	
	Zn	2.71	3.16	
	Cu	0.59	0.73	
	Cr	0.08	0.09	
	Li	0.56	0.28	
Sorghum	Na	667	870	Idris et al. (2005), Elkhier and Hamid (2008) and Nour et al (2010)
	K	463	700	
	Ca	330	440	
	Mg	464	690	
	P	434	590	
	Fe	42	90	
	Zn	477	750	
	Mn	408	700	

### 5.5 Vitamins

Cereal grains comprise vitamin E (which comprises tocotrienols and tocopherols) in the range of 0.9–4.1 mg/100 g concentrations. The intake of 100 g of cereals each day contributes 8%–34% of these vitamins. Besides, the content of riboflavin (B2), thiamine (B1), and pyridoxal (B6) vary, depending on the cereal, from 0.02 to 0.14 mg/100 g, 0.2–0.5 mg per 100 g, and 0.25–0.76 mg per 100 g, correspondingly (Hucker et al., 2012). Eventually, the amount of niacin (B3) in cereal grains is 2.7–7.6 mg/100 g, while that of folate (B9) is 0.016–0.143 mg per 100 g. Eating 100 g of cereals a day provides 20%–50% of thiamine and niacin RDA and 10%–30% of pyridoxal RDA. The effect on the RDA of grain riboflavin is much less prominent and contributes to 2%–11%. When consuming 100 g of oat or rice grains, adults comply with 5%–11% of the Recommended Dietary Allowances values of folate, 13%–29% of the Recommended Dietary Allowances by eating 100 g of barley and wheat, and maybe even up to 20%–47% by eating 100 gram of rye grains. The vitamin of grains is essential for the growth of seedlings (Hucker et al., 2012) and represents the amount of biosynthesis throughout sprouting. The variations in the vitamin of sprouted seeds are determined by the form of grain and by the steeping as well as sprouting conditions. The presence of vitamin C is typically negligible or very low in grains. Vitamin C is synthesized throughout sprouting, therefore, contributing to 5–55 mg/100 g in sprouted millet, wheat, and sorghum (Coulibaly & Chen, 2011). Thus, controlled parameters must be chosen carefully to retain this vitamin because it is among the most heat‐sensitive and light‐unstable vitamins. As a consequence of sprouting, the B complex vitamins in cereals increase dramatically and encourage the production and productivity of seedlings. Indeed, when sprouted at 25–30℃, for 3–4 days, a 1.2‐ to 5.5‐fold rise in thiamine content was observed in wheat, sorghum, and rice. Similarly, when sprouted at 17–28℃ for 4 days, the riboflavin composition in millet, barley (Hucker et al., 2012), and wheat doubled and reported for 0.07–0.25 mg per 100 g of dm. Even, when sprouted at 25℃ for 3–4 days, a 1.3‐ to 1.5‐fold rise in niacin and pyridoxal content was measured in millet, wheat, and sorghum. However, Moongngarm and Saetung (2010) noted that the amount of riboflavin, niacin, thiamine, and pyridoxine remains unchanged or even decreases for only 1 day when rice is sprouted or steeped. The authors found that even in further sprouting phases is de novo vitamin formation started and also that water‐soluble vitamins can be leached into the steep water (Moongngarm & Saetung, 2010).

### 5.6 Phenolic compounds

The effect of sprouting on total phenolic content and antioxidant activity of different cereal grains are shown in Table 2. A set of small molecules distinguished by their arrangements taking as a minimum one phenol are phenolic compounds. Phenolic compounds may be classified into various subclasses, including flavonoids, tannins, phenolic acids, quinones, coumarins, lignans, tannins, and curcuminoids, based on their chemical formulas. Likewise, phenolics primarily exist either in dissolved or an attached system in the plant. However, attached phenolic structures are produced by the transport to the cell wall of soluble phenolic compounds, which are then conjugated through ester and glycosidic bonds with cell wall organic molecules including cellulose or protein, thus leading to the creation of cell wall (Agati et al., 2012). In recent studies, it is suggested that phenolics have risen in certain sprouted grains relative to their whole‐grain equivalents. In various sprouted seeds, there is a substantial difference in total phenolic content (TPC) and sprouting which substantially absorbs TPC in bloomed grains, relative to the related raw grains. In a minority of sprouted grains, although, multiple researchers have described a decline in soluble TPC, including sprouted soybean and lentil, mung bean, and black bean. Bonded phenolics in germinated grains have not been studied as extensively as soluble phenolics, while some germinated and raw seeds produce high levels of bounded TPC. Furthermore, many studies reported that some germinated grains' bound TPC first reduces and afterward raises throughout sprouting, but bonded TPC is also demonstrated to affect steadily in many other germinated grains, including such germinated brown rice and germinated brown wheat. The amount of bonded TPC is hypothesized to be based on the breakout and conjugation rate of linked phenolics. Owing to the breakdown of their conjugates in plant cells, including the proteins and carbohydrates, bonded phenolics may be emitted from the intercellular complex mostly during the initial point of sprouting (Liu et al., 2011).

**TABLE 2:** Effect of sprouting on total phenolic content and antioxidant activity of different cereal grains

Bioactive component	Cereal	Activation temperature (°C)	Activation time (days)	Initial value	Final value	References
Antioxidant activity (AOX) **(**μmol of Trolox equiv/g of DW**)**	Rice	27 ± 2℃	6 days	1.3	1.6	Umnajkitikorn et al. (2013)
AOX (DPPH scavenging activity)	Barley	24℃	2 day	16.9	34.28	Sharma and Gujral (2010)
AOX (DPPH scavenging activity)	Wheat	35℃	3 days	7.5	12.4	Hung et al. (2011)
AOX (DPPH scavenging activity)	Corn	37℃	3 days	34.05	27.4	Ramadan et al. (2012)
AOX (DPPH Scavenging activity)	Sorghum	37℃	1 days	35.8	27.8	Ramadan et al. (2012)
AOX (DPPH scavenging activity)	Wheat	37℃	12‐hr soaking	34.4	31.08	Ramadan et al. (2012)
AOX (DPPH scavenging activity)	Corn	37℃	12‐hr soaking	32.05	27.59	Ramadan et al. (2012)
AOX (DPPH scavenging activity)	Sorghum	37℃	12‐hr (soaking)	36.28	32.06	Ramadan et al. (2012)
AOX (DPPH scavenging activity)	Common Buckwheat	20℃	6 days	14.5	88.8	Ren and Sun (2014)
AOX (DPPH scavenging activity)	Tartary Buckwheat	30℃	6 days	15.8	89.27	Ren and Sun (2014)
Total Phenolic Content (mg/g)	Oats	20℃	7 days	0.50	1.21	Tian et al. (2010)
TPC (mg/100 g)	Brown rice	28℃	2 days	72.3	86.3	Moongngarm and Saetung (2010)
TPC (mg/100 g)	Rough rice	28℃	3 days	72.3	95.6	Moongngarm and Saetung (2010)
TPC (mg/100 g)	Rye	20℃	7 days	342	446	Ti et al. (2014)
TPC (mg/100 g)	Brown rice	25℃	1 days	172.0	285.9	Ti et al. (2014)
TPC (mg/100 g)	Wheat	37℃	12 hr (soaking)	383.4	325.5	Ramadan et al. (2012)
TPC (mg/100 g)	Corn	35℃	12 hr soaking	373.3	285.5	Ramadan et al. (2012)
TPC (mg/100 g)	Sorghum	35℃	12 hr soaking	205	176.6	Ramadan et al. (2012)
TPC (mg/100 g)	Wheat	35℃	3 days	385.4	227.7	Ramadan et al. (2012)
TPC (mg/100 g)	Corn	35℃	3 days	284.5	167.7	Ramadan et al. (2012)
TPC (mg /100 g)	Sorghum	35℃	3 days	203	136.7	Ramadan et al. (2012)
TPC (µg/g FAE)	Barley	20℃	2 day	3,170	2,523	Sharma and Gujral (2010)
TPC (µg/g FAE)	Wheat	25℃	24 hr steeping	2,320	2019	Hung et al. (2011)

### 5.7 Flavonoids

In germinated grains including kaempferol, rutin, quercetin, luteolin, and their O‐glycosides, numerous different flavonoid aglycones, as well as their O‐glycosides, are known. The key ones in various germinated grains, however, may be different basic flavonoids. In germinated mung beans, broccoli, and sunflower, apigenin has also been reported (Pająk et al., 2014). In germinated buckwheat, adzuki bean, climbing bean, green mung bean, sesame bean, and rice bean Catechin has been found (Ha et al., 2017). Sprouted climbing bean, adzuki bean, rice bean, mottled cowpea, and sesame bean have been identified with epicatechin (Gan et al., 2016). In sprouted dark beans, hesperidin 7‐glucoside and naringenin 7‐glucoside were found (López‐Cervantes et al., 2013). In mung beans, dark beans, radish, sunflower, broccoli, and buckwheat, Kaempferol has also been identified. Many other derivatives of kaempferol, including kaempferol 3‐O‐rutinoside, kaempferol 3‐glucoside, and kaempferol 3‐rutinoside acylate, kaempferol dirhamnosyl‐galactopyranose, kaempferol apiofuranosyl rhamnopyranosyl‐galactoside, kaempferol glucuronide, were also identified in several germinated grains, which including sprouted mung beans, common beans, dark beans, buckwheat, etc (Paucar‐Menacho et al., 2018). In germinated radish, mung beans, buckwheat, sunflower, broccoli, and sesame, luteolin has been found (Ha et al., 2017). In germinated buckwheat and dark bean, myricetin was found and its derivative myricetin 3‐glucoside was discovered in the germinated dark bean. Quercetin has been constituted in germinated dark bean, mung bean, broccoli seed, radish seed, buckwheat and sunflower seed and many quercetin derivative products which including isoquercitrin, quercetin, quercetin 3‐rutinoside, quercetin 3‐O‐glucoside, quercetin 3‐glucoside acetate, quercetin 3‐rhamnoside, quercetin rhamnosyl glucuronide, quercetin dirhamnopyranosyl‐galactopyranoside, and quercetin 3‐glucoside acetate. In sprouted mug beans, kidney beans, and buckwheat, Rutin was reported. Common flavonoids occur predominantly insoluble compounds of germinated grains, but many of these have also been found in the bonded compounds of sprouted grains, including apigenin, kaempferol, luteolin, and quercetin, indicating that flavonoids may even be included in the cell wall structure.

## 6 FOODSTUFFS FROM SPROUTED GRAINS

Consumers today are particularly concerned about food items with high bioavailability, outstanding sensory characteristics, and extended shelf life. The health benefit of biologically stimulated or sprouted grain has been highlighted by many studies, and if these grain can be used in traditional foods, it will increase metabolism, improve immunity, substitute for mineral and vitamin deficiency, and normalize the combination of acid and base. The development of biomaterial compounds during the biological activity of grains contributes to the enhancement of organoleptic properties because of texture loosening and increased flavors in oat, finger millet, barley, and rye, although problems typically associated with preparing brown rice have also been addressed. In addition to increasing the nutritional content of bread with higher Vit C, β glucan, vitamin E, dietary fiber, and folate content in wheat as well as Egyptian pita bread, the use of biologically modified grains as an additive was found. Products prepared from sprouted cereal grains are shown in Table 3.

**TABLE 3:** Products prepared from sprouted cereal grains

Germinated grain	Product in which grains are incorporated	References
Wheat	Bread	Iordan et al. (2013)
Brown rice	Bread	Morita et al. (2007)
Quinoa and oat	Bread	Mäkinen et al. (2013)
Wheat, Barley, Rye	Bread	Rakcejeva et al. (2005)
Buckwheat	*Natto* and *Miso* paste	Miyake et al. (2006)
Wheat	Yoghurt	Zagorska et al. (2010)
Oat	Bread	Rakcejeva et al., (2005)
Brown rice	Cookies	Chung et al. (2014)
Sorghum	Bread	Phattanakulkaewmorie et al. (2011)
Maize	Cookies	Adedeji et al. (2014)
Finger millet	Bread	Bhol and Bosco (2014)
Brown rice	Noodles	Chung, Cho, & Lim, et al. (2012) and Chung, Cho, Park et al. (2012)

Iordan et al. (2013) stated that the use of biologically modified preparations including sprouted wheat enhanced the overall sensory attributes of bread at a 15% bread level. They found that the addition of less barley of biologically modified rye, wheat, and hull improved the vitamin content throughout the bread relative to control samples and reduced the value of depletion of bread baking. The assessment of sensory attributes shows that the use of biologically modified wheat and barley seed as an ingredient in producing bread that enhances its taste and texture with a noticeable effect on bread crumb, coloring, and porosity, and the panelists also recommended some other bread products the wheat flour with biologically modified wheat and hull‐less barley seed. Oat, as well as quinoa malts, have also been introduced into a gluten‐free formula or bread‐based on rice and potato (Mäkinen et al., 2013).

They also found that this characteristic of bread may also be delivered as frozen bread which would have a longer life span, or may be sold as a food service commodity that, as usually done in some major supermarkets, may be made‐to‐order or made fresh every day. Morita et al. (2007) also make use of pre‐germinated brown rice and various bread processing additives together. The bread comprising sprouted sorghum (50:50) had good texture (hardness) than any of those comprising higher phenolic substances of ungerminated sorghum bread and malted sorghum bread. Consequently, the crust and also crumbs of sorghum blended gluten‐free bread were tougher and less flexible than that of wheat bread; thus, some improvements would be required to obtain the optimal characteristics of sorghum gluten‐free bread, such as incorporating some hydrocolloids and emulsifiers. Chung et al. (2014) documented that replacing wheat flour, sugar snap biscuits with germinated brown rice (GBR) flour increased their physical characteristics with low water content and a high spreading factor than that of containing raw GBR flour and also delayed storage moisture loss and hardening. They also revealed that cookies of extremely high quality, as well as adequate nutrition, could be produced by replacing wheat flour with high‐temperature GBR flour partially or entirely.

Bhol and Bosco (2014) also noted that bread containing 20% malted finger millet has better sensory and textural properties that enhance its nutritional traits, that is, protein, dietary fiber, and mineral content, thus demonstrating the probability of using millets to boost bread's nutritional quality. Miyake et al. (2006) noted that sprouted buckwheat is an efficient, acceptable, and useful low‐ and a nonallergenic response for the manufacture of natto (soba natto) and miso paste (soba miso paste) and can substitute traditional soybean products. Adedeji et al. (2014) used sprouted maize flour in cookie formulation and found that germination occurred in biscuits with improved gelatinization at various baking temperatures, with the best resulting sample germinating for 72 hr. The substitution of 20% of yogurt with genetically modified wheat grain chips improved sensory attributes and boosted protein as well as dietary fiber quality (Zagorska et al., 2010). Compared to normal yogurt, it also enhanced the product's taste and texture with greater acceptability as well as palatability. Therefore, the inclusion of wheat grain chips may be a successful opportunity for dietary fiber to enrich the nutritional value of yogurt.

## 7 CONCLUSION

Consumers today are particularly concerned about food items with high bioavailability, outstanding sensory characteristics, and extended shelf life. The health benefit of biologically stimulated or sprouted grain has been highlighted by many studies, and if these grain can be used in traditional foods, it will increase metabolism, improve immunity, substitute for mineral and vitamin deficiency, and normalize the combination of acid and base. During germination, many biochemical modifications occur, impacting product possessions such as shape, bioactivity, consistency, flavor, and digestibility. Germination induces enzymes to split up carbohydrates, lipids, and proteins into basic compounds and stimulates protease involved in protein degradation, thus enhancing the bioavailability of nutrients. Starch and proteins are activated and decomposed by hydrolytic enzymes, resulting in a rise in oligosaccharides and amino acids in oat, wheat, barley, and rice.

## CONFLICT OF INTEREST

There is no conflict of interest by the authors.

## AUTHOR CONTRIBUTIONS

**ALI IKRAM:** Writing‐original draft (equal). **Farhan Saeed:** Supervision (equal). **Muhammad Afzaal:** Writing‐review & editing (equal). **Ali Imran:** Writing‐review & editing (equal). **Bushra Niaz:** Visualization (equal). **Tabussam Tufail:** Formal analysis (equal). **Muzzamal Hussain:** Data curation (equal); Methodology (equal). **Faqir Muhammad Anjum:** Project administration (equal).
